# Supplementary material for: Rapid turnover of life-cycle-related genes in the brown algae
Source: Genome Biol. 2019 Feb 14;20:35. doi: 10.1186/s13059-019-1630-6 (PMC6374913; doi:10.1186/s13059-019-1630-6)
Supplement: Supplementary file 3 — Protein alignments, corresponding Gblocks html file and CDS sequences for the lineage conserved gametophyte-biased genes. (ZIP 2109 kb) [file 13059_2019_1630_MOESM3_ESM.zip › Con_GA_alignments/Ec-00_001220.aa_ali.fasta-gb.htm]

Ec-00\_001220.aa.fasta.trans.aa\_ali.fasta


## Gblocks 0.91b Results

Processed file: **Ec-00\_001220.aa.fasta.trans.aa\_ali.fasta**  
Number of sequences: **2**  
Alignment assumed to be: **Protein**  
New number of positions: **534** (selected positions are underlined in blue)

```
                         10        20        30        40        50        60
                 =========+=========+=========+=========+=========+=========+
Ec-00_001220     ------------------------------------------------------------
MSTRG.51699.1    XFSDEDAYAFREGGNVDAGAHYPSNDSGVDATTTGGIGPGAEPPVAIAFHAFAHDEPCTD
                                                                             


                         70        80        90       100       110       120
                 =========+=========+=========+=========+=========+=========+
Ec-00_001220     ------------------------------------------------------------
MSTRG.51699.1    AACGGSKSTIEQGTETIPLGVSCSDREQMRGLSVERGDETRGDEKRLFGGKQDIAVWPPT
                                                                             


                        130       140       150       160       170       180
                 =========+=========+=========+=========+=========+=========+
Ec-00_001220     ------------------------------------------------------------
MSTRG.51699.1    STRTSNEGEADTLPDSFYLSDEYGPEGASTGRNHHDDDDNDGSTNGRREPLSIGSPTEAG
                                                                             


                        190       200       210       220       230       240
                 =========+=========+=========+=========+=========+=========+
Ec-00_001220     ------------------------------------------------------------
MSTRG.51699.1    CGVIRGGARPSSGRYSVENSNDRDAPSGFQRRQEEKSADMFGKSSDGSDYWSSSSETLHH
                                                                             


                        250       260       270       280       290       300
                 =========+=========+=========+=========+=========+=========+
Ec-00_001220     ------------------------------------------------------------
MSTRG.51699.1    QPRRLPQPRFSRRRARQNSRRAPLDTTQRSSRIAPSEDLASPTDLTDVETEGGRRHRRTS
                                                                             


                        310       320       330       340       350       360
                 =========+=========+=========+=========+=========+=========+
Ec-00_001220     ------------------------------------------------------------
MSTRG.51699.1    CRGSVKHRSPPRRHPRGKEDSSCNRRGRRGVSGYRKRDLRREALTETESMCGEPNDELEE
                                                                             


                        370       380       390       400       410       420
                 =========+=========+=========+=========+=========+=========+
Ec-00_001220     ------------------------------------------------------------
MSTRG.51699.1    EVAKLRRENEELKRRQEGTTRLSDQGFDVEPWKNGESIDGCFAKRDSSRRATRRRDDTFD
                                                                             


                        430       440       450       460       470       480
                 =========+=========+=========+=========+=========+=========+
Ec-00_001220     ------------------------------------------------------------
MSTRG.51699.1    IEGRSQRLSREGKARVGQQELQHEHRRRSRSIALTSHLSGVEASTGRSSTRRRQGVPTWT
                                                                             


                        490       500       510       520       530       540
                 =========+=========+=========+=========+=========+=========+
Ec-00_001220     ------------------------------------------------------------
MSTRG.51699.1    EFVSKSNSSPAAKSPMGSVGPHSWASRSSENAYPRFDLSLPRVLVEGGYLWKIPCHNAGT
                                                                             


                        550       560       570       580       590       600
                 =========+=========+=========+=========+=========+=========+
Ec-00_001220     -------MKPAEGLLTTASGRLVVRPTAGGGGGSG--VCGREADRGNESSRAGGGDKGAA
MSTRG.51699.1    PKRRWFQIKPADGLLTTASGHLVVRPTSAGGVGAGGSAVGRRKIDSAVSRRSEDGEESTG
                         ###########################    ################     


                        610       620       630       640       650       660
                 =========+=========+=========+=========+=========+=========+
Ec-00_001220     PSIPMMRLHAAWPLTFIWVDPERDLKRFPPREIVVEEIVDLARGHKTPAFWQQASHRGIH
MSTRG.51699.1    -SVPMMRLRAAWPLALIWVDPERDLKKSPPREMGIEEVMDLARGHKTPAFWQQAAHRGIH
                  ###########################################################


                        670       680       690       700       710       720
                 =========+=========+=========+=========+=========+=========+
Ec-00_001220     TLPDPKLCFSLVGHDRSLDLAAESLPEAREWVCALARVALMAKRGGLVEAAGDAVAPLLP
MSTRG.51699.1    TLPDPKLCFSLIGSKRTLDLAAEGLLEAKEWVLALARVALMVDRGGLAAANG--YAAVCS
                 ####################################################   #####


                        730       740       750       760       770       780
                 =========+=========+=========+=========+=========+=========+
Ec-00_001220     HRFWPEPAASPAGPDLEENMSGERLIDVDHEVD--SRVGGQRQ-SDRAATALNTEVSRHE
MSTRG.51699.1    PRSQVEPATTPHSSDSEE-LSSARLRDNHGGGDEISHVGLQRRPSKKSGKPLR---KKEE
                 ##################  #############                           


                        790       800       810       820       830       840
                 =========+=========+=========+=========+=========+=========+
Ec-00_001220     VSLRSDADLSSEIPKVFGEGGAWSVDTIHAWRRRLFPAVRRGDIAAVLAIFDQGCPVDIA
MSTRG.51699.1    FCTRSEENSAHVKSGVLGGGRASSVDTTRSWRQRIFPAVSRGDVASVLAMFDQGCPVDLV
                                #############################################


                        850       860       870       880       890       900
                 =========+=========+=========+=========+=========+=========+
Ec-00_001220     QTGTGDTPMLLACRLGDVGMVRECLRRGSRNDPHPAFGQTALQAAVASGQEACARLILET
MSTRG.51699.1    HEETGDTPMLLACRLGDIGMVTECLRRGARNDPHPNFGQTALQAAVAAGREACARLLLET
                 ############################################################


                        910       920       930       940       950       960
                 =========+=========+=========+=========+=========+=========+
Ec-00_001220     AAPSRSDAVVSNHKDPNKETPLHVACRRGYDGIVEALLHHGADLRAVDRKGNTPLHGAAG
MSTRG.51699.1    AAPSRSDVVVSNHKDPNKDTPLHVACRRGYRGIVEALLHHGGDLRVANKKGNTPLHGAAG
                 ############################################################


                        970       980       990      1000      1010      1020
                 =========+=========+=========+=========+=========+=========+
Ec-00_001220     SGHAGALGSLLDAGGDVVLEERNARGDRALHTAAAPGHIACVELLLGTAAEPDVPNADGQ
MSTRG.51699.1    CGHAAVLASLLDAGGDAVLEEKNLRGERAMHTAAASGHIACVELLLGTAAEPDIPRTDGQ
                 ############################################################


                       1030      1040      1050      1060      1070      1080
                 =========+=========+=========+=========+=========+=========+
Ec-00_001220     TPFALASRGGHLAVTRLIRKYAPAAGTPSGGDAQFQAHRRANEGGGSATADALPRPHSSG
MSTRG.51699.1    TPFMLASRGGHLAVTRLIRRYAPAAGTPSGGDAQFQTHQRANTGRQSATADALPRPHASG
                 ############################################################


                       1090      1100      1110      1120      1130      1140
                 =========+=========+=========+=========+=========+=========+
Ec-00_001220     SASN----PTP-DHRSRDLEHRCYSAAGLGHQQFEDLNDPTSCRQPVYTSGADSSDRDLL
MSTRG.51699.1    PASTAIGIPSPTAHQDRDLENRFYSAAGLEHR------EPPSSETALTY-----------
                 ###          ##################                             


                       1150      1160      1170      1180      1190      1200
                 =========+=========+=========+=========+=========+=========+
Ec-00_001220     RSTSEQDRRGLYARGGELHNTKATPPQSTVEEDVISPASSASAPVQGRHGSLFDGLQVYV
MSTRG.51699.1    ---TGNERMGC-AR-----------LRS---------APNASAPLP--HGSLFDGLQVYV
                                                                 ############


                       1210      1220      1230      1240      1250      1260
                 =========+=========+=========+=========+=========+=========+
Ec-00_001220     VGGRQPLGSQHS--CAAPGNVS-ALARVPVSARTMVLDSHRLSTASHHHQRREEQQQQHQ
MSTRG.51699.1    VGGRQPLGSQHASTAAKAGIVSKPSPQAPTSARTMILEGRCLSAS--------LQQQQQQ
                 ###########                 ###############                 


                       1270      1280      1290      1300      1310      1320
                 =========+=========+=========+=========+=========+=========+
Ec-00_001220     NYYPSNQHTSYNSSLSTASFEGLDPNWRDNVLSAPSTVAPSPPTPVLPPEDDRRPSCDST
MSTRG.51699.1    ------------------------------------------------------------
                                                                             


                       1330      1340      1350      1360      1370      1380
                 =========+=========+=========+=========+=========+=========+
Ec-00_001220     PLLSAVPSTATTNIGDGTSVVGVPGSARALARMIHFNSTNGRDENEISRRVLGRGTSRSG
MSTRG.51699.1    ------------------------------------------------------------
                                                                             


                       1390      1400
                 =========+=========+=======
Ec-00_001220     ATAGQEAVEIEESEKEGWGGENSRFSC
MSTRG.51699.1    --------------------------X
```

```
Parameters used
Minimum Number Of Sequences For A Conserved Position: 2
Minimum Number Of Sequences For A Flanking Position: 2
Maximum Number Of Contiguous Nonconserved Positions: 8
Minimum Length Of A Block: 10
Allowed Gap Positions: None
Use Similarity Matrices: Yes
```

```
Flank positions of the 9 selected block(s)
Flanks: [549  575]  [580  595]  [602  712]  [716  738]  [741  753]  [796  1083]  [1094  1111]  [1189  1211]  [1229  1243]  

New number of positions in Ec-00_001220.aa.fasta.trans.aa_ali.fasta-gb:  534  (37% of the original 1407 positions)
```
